# Ab-Externo XEN Gel stent implantation effectively treated refractory glaucoma with prior failed shunt tube

**DOI:** 10.1186/s12886-024-03648-7

**Published:** 2024-08-30

**Authors:** Anny M.S. Cheng, Shailesh K. Gupta, Geetha G. Vedula, Jackson Saddemi, Victor Wang, Rita Vartanian, David T.Y. Yang, Aarup A. Kubal

**Affiliations:** 1 Department of Ophthalmology, Broward Health, Fort Lauderdale, FL USA; 2 Specialty Retina Center, Deerfield Beach, FL USA; 3 Department of Ophthalmology, Florida International University, Herbert Wertheim College of Medicine, Miami, FL USA; 4 Your Eye Specialists, 1776 N. Pine Island Rd., Suite 214, Plantation, FL 33322 USA; 5 Burnett School of Biomedical Sciences, University of Central Florida, Orlando, FL USA; 6 College of Biological Science, University of California, Davis, Sacramento, CA USA

**Keywords:** Glaucoma drainage device, Minimally invasive Glaucoma surgeries, Shunt tube, Tenonectomy, XEN Gel Stent

## Abstract

**Purpose:**

To assess the efficacy of a gelatin stent (XEN 45 Gel Stent; Allergan) implant in advanced glaucoma eyes that have failed prior aqueous shunt implantation.

**Methods:**

We retrospectively reviewed 6 patients with refractory glaucoma, defined as persistently high IOP (> 21 mmHg) despite taking at least 3 IOP-lowering medications subsequent to undergoing a glaucoma drainage device (GDD) with or without a second GDD or cilioablative procedure. Eyes with previous failed GDD underwent subconjunctival 0.3 cc (0.4 mg/ml) mitomycin C, tenonectomy, and placement of an ab- externo XEN stent. The outcome measures included change in IOP and the number of glaucoma medications. Success was defined as patients achieving an IOP ≤ 18 mmHg with a percentage reduction of 25% or 15 mmHg and 40% mean IOP reduction from baseline while taking the same number or fewer medications.

**Results:**

All six eyes with age of 77.6 ± 7.82 years who underwent XEN implantation following previous GDD surgery had primary open-angle glaucoma. The IOP decreased significantly from 32.33 ± 5.99 to 12.67 ± 3.27 mmHg (*p* < 0.001) with a follow-up of 13.9 ± 2 (11.7–16.7) months. Visual acuity and visual field remained stable after XEN placement. Compared to the baseline number of medications of 4.2 ± 0.8, all medication was discontinued except in one eye on two drops at the end of the follow-up. The overall surgical success rate was 100%. No complications, needling, or additional procedures were required.

**Conclusion:**

This study described successful implantation of the XEN stent following failed GDD. XEN Gel stent implantation associated with mitomycin C and tenonectomy can be considered a viable surgical option for patients with a history of previously failed tube shunt requiring further IOP lowering.

## Introduction

Glaucoma is a progressive neurodegenerative optic neuropathy and the leading cause of irreversible blindness worldwide [1]. Intraocular pressure (IOP) is the major modifiable risk factor for the development and progression of glaucoma [2]. To date, a reduction in IOP is the only proven method to slow the progression of visual field loss and preserve vision [3]. Implantation of a Glaucoma drainage device (GDD), also known as a tube shunt, is used to provide an alternate pathway for aqueous humor to drain usually from the anterior chamber [4], hence lowering IOP in patients that are at high-risk, after failed trabeculectomy, or as a primary procedure [5–7]. However, even with a patent and functional tube shunt in addition to maximal medical treatment, IOP may still be inadequately controlled, resulting in the progression of vision loss. Furthermore, a GDD is associated with the risk of subconjunctival fibrosis and other potential surgical complications over time, which may result in inadequate IOP control and require further procedures [8]. Recently, minimally invasive glaucoma surgeries (MIGS) have been developed to reduce surgical complications, and XEN^®^ 45 gel stent (Allergan PLC, Irvine, CA, USA) is one of these new treatment options [9].

The XEN gel stent is a flexible, hydrophilic 6-mm tube with a lumen size of 45 microns [10]. The tube’s flow resistance of around 6–8 mmHg is designed to prevent hypotony based on the concepts of laminar fluid dynamics. The implant’s substance, porcine collagen-derived gelatin cross-linked with glutaraldehyde, is non-inflammatory [11]. The XEN stent targets the subconjunctival space for aqueous drainage, which has demonstrated efficacy in reducing IOP, with a 1-year IOP reduction ranging from 29 to 46% in patients with refractory glaucoma (reviewed in [10] ). Few studies to-date have demonstrated the efficacy of XEN stent in patients with a previous unsuccessful GDD implantation. A prospective multicenter nonrandomized trial of 45 eyes with XEN140 stent placement without intraoperative mitomycin C (MMC), in which 2 eyes had prior tube shunt surgery, showed a mean IOP reduction of 36.4% and a decrease in the number of medications from 3 to 1.3 [12]. A retrospective study of 18 eyes with an ab-externo XEN stent with a prior GDD implant (*n* = 8) or trabeculectomy (*n* = 13) showed an average of 25% IOP reduction [13]. A recent case report describes a case of refractory open-angle glaucoma with failed Baerveldt glaucoma implant (BGI) and trabeculectomy that was successfully treated with ab- externo XEN gel implant where the IOP remained in the 15–18 mm Hg range without glaucoma medication [14]. Another retrospective study in Poland involving 43 patients who underwent ab-interno or ab-externo XEN implantation with previous surgeries, of which 8% had Ahmed valve implantation, reported a 27% decrease in IOP from 25.4 ± 7.8 mmHg to 17.5 ± 5.5 mmHg [15] .However, There remains limited published data directly addressing XEN stent success in patients who have failed prior GDD placement. Our study aims to evaluate the efficacy and safety of XEN Gel stent implantation with MMC in eyes that required additional IOP-lowering treatment after GDD implantation.

## Methods

This retrospective case series was conducted at Your Eye Specialists (YES) in Florida. The Broward Hospital District Health Ethics Committee granted the Institutional Review Board’s approval for the protection of human subjects. All research adhered to the principles of the Declaration of Helsinki and the Institutional Review Board determined that informed consent for this study could be waived.

We included patients with refractory glaucoma, defined as a previous failure of GDD with or without a second GDD or cilioablative treatment and persistently high IOP (> 21mmHg) despite taking at least three IOP-lowering medications. Exclusion criteria included a shallow anterior chamber and angle-closure glaucoma, the presence of clinical inflammation or infection within 30 days prior to surgery, corneal opacity that prevented intraoperative viewing of the anterior chamber, and conjunctival scarring in the target quadrant. The type of glaucoma, history of surgery, IOP, medications (a fixed combination agent was counted as two medications), visual acuity (VA), visual field (VF) and complications were recorded. The main outcome measures included changes in IOP, VA, VF defect, the number of glaucoma medications, and success rates. Success was defined based on the criteria previously outlined by the European Glaucoma Society [16]. This included patients who achieved an IOP ≤ 18 mmHg with a percentage reduction of 25% or 15 mmHg and 40% mean IOP reduction from baseline while taking the same number or fewer medications [16]. Failure was defined as additional glaucoma surgery, an IOP reduction of < 20% IOP with maximum tolerated glaucoma medications, or an irreversible loss of light perception [16].

Despite intensive medical treatment, our patients had either uncontrollable IOP and progressive VF loss. We performed traditional surgery based on the findings of the Primary Tube Versus Trabeculectomy (PTVT) Study, which indicated a higher likelihood of severe complications that cause vision loss and/or requiring further surgery for complication management after trabeculectomy compared to tube shunt surgery [17]. Hence, all our patients had primary tube shunt surgery instead of primary trabeculectomy.

### Surgical technique

**Fig. 1:**
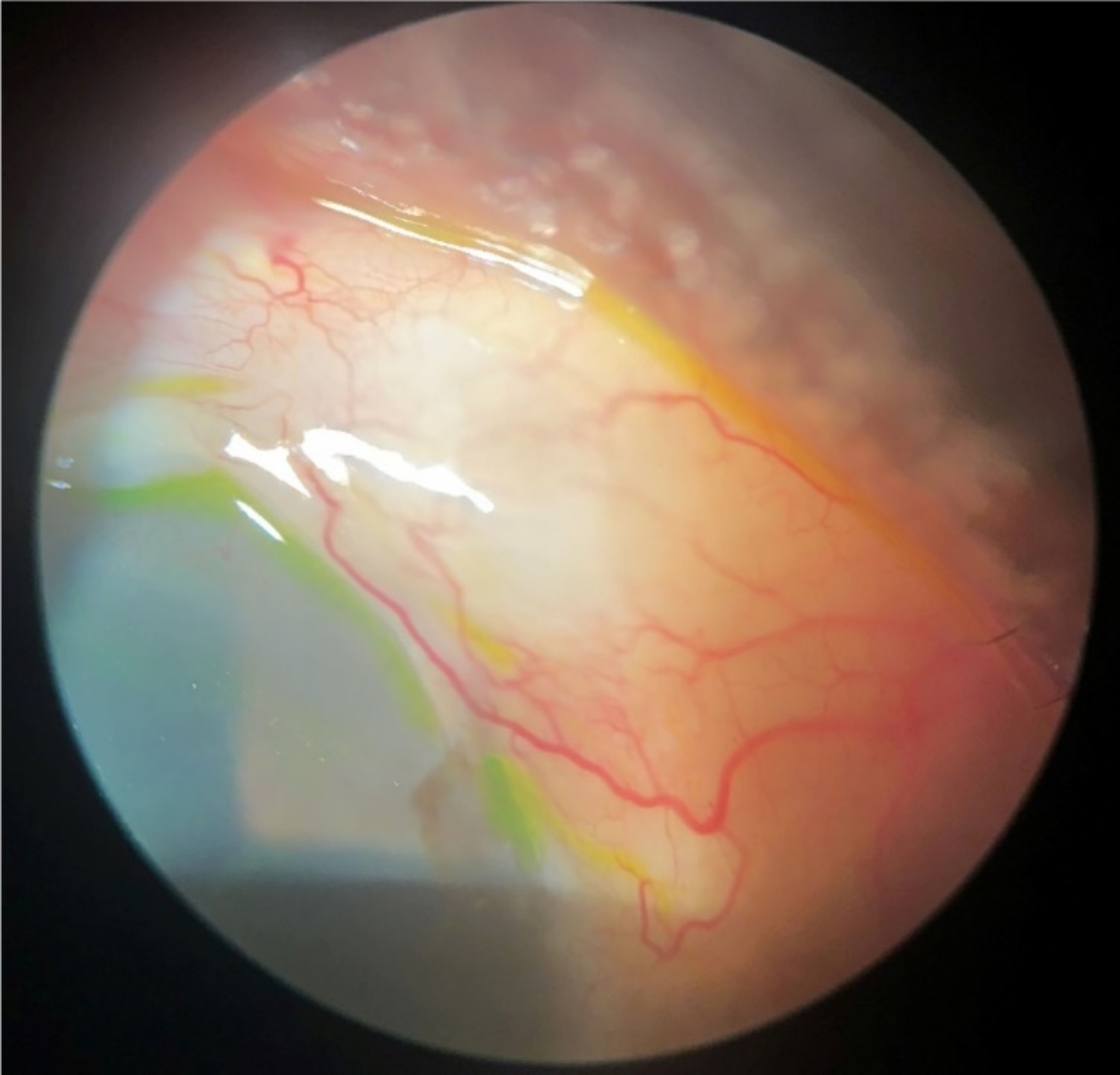
Functional bleb of XEN Stent. An elevated subconjunctival bleb is drained by a XEN stent located in the superonasal quadrant

The surgeries were performed by a single surgeon (AAK). The surgical technique, including use of an ab-externo open conjunctiva approach with a relatively large amount of mitomycin C, was optimized over hundreds of cases in the surgeon’s patient population. The procedure was performed under local anesthesia with a retrobulbar block. A 6–0 vicryl traction suture was placed at the superior corneal margin to depress the eye. 0.3 ml of 0.4 mg/ml of MMC was injected with a 30-gauge needle in the subconjunctival space and directed posteriorly to obtain a bleb in the superonasal quadrant. The cornea was then irrigated extensively to wash away any MMC remaining. A microperitomy was performed, the subconjunctival space was irrigated, and the tenon was dissected and excised from the sclera. The intended area of XEN placement in the superonasal quadrant, 2 mm posterior to the limbus, was marked. All patients had XEN stents positioned in the superonasal quadrant (Fig. 1), directing them away from their primary superotemporal GDD. The XEN45 injector needle was passed through the marked sclera and visualized emerging into the anterior chamber. Once the tip of the needle was visible, the stent was gently delivered by advancing the slider. The needle housing the implant was retracted without drawing the XEN implant back. The stent placement left 3.0 mm of exposed XEN implant in the subconjunctival space and 1.0 mm in the anterior chamber. A XEN implant positioned anterior to the trabecular meshwork without trauma to the cornea or iris was then verified by gonioscopy. The conjunctiva was closed with two 8–0 vicryl wing sutures and confirmed to be leak-free. 

Antibiotic treatment with moxifloxacin four times daily was continued for the first week, along with topical corticosteroid treatment with Prednisolone 1% four times daily, tapered weekly and discontinued in one month. All anti-glaucoma medications were discontinued on the day of surgery and resumed only if the target IOP was not achieved following surgery, in accordance with the Advanced Glaucoma Intervention Study rule [18]. 

Statistical analysis was performed using a one-tailed paired t-test to compare data in baseline and post-operative visits with SPSS statistical software (V. 22.0. IBM Corp, Armonk, NY). Mann-Whitney Rank Sum test was used if failed Normality test (Shapiro-Wilk) in IOP test. The best corrected visual acuity (BCVA) was transformed into the logarithm of the minimal angle of resolution (log MAR) for the purpose of statistical analysis. Descriptive statistics are presented as mean ± standard deviation and statistical significance was set at *P* < 0.05.

## Results

Six eyes of six patients, five male and one female, who underwent ab-externo implantation of XEN between August 2022 and May 2023 following previous GDD surgery were reviewed. Three patients were black, two were white and one was Asian. The mean age of the patients was 77.6 ± 7.82 (68–84) years. All six patients had primary open angle glaucoma (POAG). Three eyes were pseudophakic while three eyes were still phakic.

Prior to GDD implantation, two eyes underwent selective laser trabeculoplasty (SLT) and one eye underwent cataract surgery and endoscopic cyclophotocoagulation (ECP) (IRIDEX Corp., Mountain View, CA). Before XEN implantation, one eye received cyclophotocoagulation (CPC), one eye received combined ECP/OMNI (Sight Sciences, Inc. Menlo Park, CA, USA), and one eye underwent repeated GDD insertion after the initial GDD placement.

Most patients had a long follow-up period between GDD and XEN placement, with a mean follow-up time of 3.7 ± 2.5 (0.9–7.2) years. For GDD implantation, four eyes had an Ahmed valve, one eye had cataract surgery combined with a Baerveldt glaucoma drainage implant, and one eye had an initial Baerveldt implant and a repeat Baerveldt device when the IOP target was not met. Most patients (*n* = 5) underwent a stand-alone XEN stent implantation, but one patient had a combined cataract and XEN stent procedure. The average duration of follow-up after the XEN implant was 13.9 ± 2 (11.7–16.7) months.

Following GDD but before XEN placement, the mean BCVA decreased by 0.58 ± 0.78 logMAR (reduced from 0.32 ± 0.16 to 0.89 ± 0.79 logMAR, *p* = 0.06), while the BCVA remained stable after XEN stent treatment (improved from 0.89 ± 0.79 to 0.75 ± 0.76 logMAR, *p* = 0.5). Similarly, the VF worsened with mean deviation (MD) loss from  -16 ± 5.9 to -22.9 ± 6 dB before XEN placement (*p* = 0.08), but it stayed unchanged after XEN placement (-22.9 ± 6 to -24.3 ± 5.3, *p* = 0.5).

The mean IOP before GDD placement was 38.17 ± 9.81 mmHg, which decreased to 32.33 ± 5.99 mmHg (baseline IOP) before the XEN implant. The IOP decreased significantly from 32.33 ± 5.99 mmHg at baseline to 12.67 ± 3.27 mmHg (*p* < 0.001) after implantation. The reduction in mean IOP was 19.67 ± 7 mmHg (59%) after XEN implantation. At the end of the follow-up period, all medication was discontinued except one eye on two drops. Compared to the baseline number of medications of 4.2 ± 0.8, the reduction was 3.8 ± 0.8. Overall, the surgical success rate was 100% as all patients achieved reduction of IOP 59% from baseline and on significantly fewer medications. (Table 1)

**Table 1:** Demographic and clinical data of patients

Case	Age (Yr)	Gender (M/F)	Race	Eye (OD/OS)	Med History	Prior glaucoma Tx	Pre-GDD IOP (mmHg)	GDD	Post-GDD Tx	Duration from GDD to XEN placement (yrs)	Baseline IOP (mmHg)	Pre-XEN Number of med	XEN Standalone or Combined Surgery	Follow up (months)	1 day Post-XEN IOP (mmHg)	1 week Post-XEN IOP (mmHg)	1 month Post-XEN IOP (mmHg)	3 months Post-XEN IOP (mmHg)	6 months Post-XEN IOP (mmHg)	12 months Post-XEN IOP (mmHg)	Final Post-XEN IOP (mmHg)	Reduction in IOP (mmHg)	Post-XEN Number of Med
1	83.3	M	Black	OD	-	-	42	AHMED	-	7.2	37	4	XEN	15.3	15	10	12	16	14	14	14	23	0
2	81.8	M	White	OD	CAD, HTN, CHO	-	20	AHMED	CPC	1.1	36	4	XEN	11.7	17	14	12	16	14	16	16	20	0
3	67.6	M	Black	OD	DM, HTN	cataract, ECP	46	BGI	BGI	6	21	5	XEN	12.3	6	13	14	15	12	17	15	6	0
4	67.6	F	White	OS	HTN, CHO	SLT	40	AHMED	OMNI + ECP	0.9	33	4	cataract + XEN	16.7	10	9	10	16	13	11	13	20	0
5	84.3	M	Black	OD	DM	SLT	35	cataract + BGI	-	3.6	36	5	XEN	14.9	13	9	14	9	20	15	11	25	2
6	81	M	Asian	OS	Arthritis	-	46	AHMED	-	3.6	31	3	XEN	12.8	5	10	11	10	7	9	7	24	0

Abbreviations: BGI: Baerveldt glaucoma drainage; CAD: cardiovascular disease; CHO: hypercholesterol; CPC: cyclophotocoagulation; DM: diabetics mellitus; ECP: endoscopic cyclophotocoagulation; F: female; GDD: Glaucoma drainage device; HTN: hypertension; IOP: intraocular Pressure; M:male; mmHg: millimeters of mercury; Med: medications; OD: right eye; OS: left eye; SLT: selective laser trabeculoplasty; Tx: treatment; Yr: years old; yrs: years; - : Nil

There were no intraoperative complications such as Descemet membrane detachment, iris injury, iridocorneal contact, lens contact, vitreous loss, hyphema, or flat anterior chamber. No cases of hyphema, choroidal effusion, suprachoroidal hemorrhage, or maculopathy were reported postoperatively. The only postoperative adverse event noted was that one eye (case 3) experienced transient hypotony (IOP < 6 mm Hg). It resolved without sequelae and did not require surgical intervention.

## Discussion

Despite the demonstrated effectiveness of XEN implants in patients with refractory glaucoma, (reviewed in [10, 19, 20]), the direct therapeutic effects of XEN implants in previously failed GDD patients are still poorly studied. Our results were comparable to multiple studies for ab-interno XEN in eyes with previous failed glaucoma surgery, although subgroup-specific analysis for prior GDD groups is not available [10, 12, 15] .This study showed that all our patients with prior failed GDD achieved an average of 59% IOP reduction from baseline (32.33 ± 5.99 mmHg to 12.67 ± 3.27 mmHg) with significantly fewer medications (reduction of 3.8 ± 0.8) and a reduced mean IOP of 19.67 ± 7 mmHg after ab-externo XEN implantation. Visual acuity and visual field defect remained stable after XEN placement. Similar findings were described in a Canadian case series that reported eyes with previous Ahmed (*n* = 5) or Baerveldt (*n* = 2) drainage receiving a XEN stent, which effectively reduced IOP from 23.9 ± 5.3 mmHg to 14.0 ± 5.3 mmHg, and the number of glaucoma medications was reduced from 4.3 ± 1.3 to 1.6 ± 1.6 at 1-year follow up [21]. We only included POAG patients and did not include patients with other types of glaucoma, which may explain why our IOP reduction appears to be different from others [21] despite a recent study finding no significant difference in the efficacy (> 25% IOP reduction) of XEN stents between POAG and other glaucoma subtypes [22] .

Intriguingly, we had a patient who had two GDD placements and whose IOP control remained suboptimal until XEN implantation. Results of the tube versus trabeculectomy (TVT) study favor implanting a GDD in patients with previous unsuccessful surgery and refractory glaucoma [23]. A recent pooled analysis of prospective multicenter randomized clinical trials revealed, however, that the cumulative probability of GDD failure at 5 years is as high as 38.3% [24]. An American study of 1945 patients with POAG treated with incisional surgery (including tube shunt or trabeculectomy) reported that more than one-fourth underwent additional procedures within 5 years to address primary surgical failure [8]. Of these, 11.6% and 27.1% of primary GDD patients (*n* = 551) have experienced failure at 1 year and 5 years follow up, respectively, and the five-year reoperation rate was 14.0%. Regarding the additional procedure, a retrospective case-control study of 18 eyes with ab-externo XEN stent, of which 8 eyes had primary GDD, demonstrated noninferior IOP reduction and a superior safety profile in comparison to 25 of 36 eyes with a second GDD implant, of which 16 eyes had primary GDD [13]. However, a study has not yet been conducted to assess the effectiveness of the XEN stent compared to a second GDD in patients who had previously failed GDD implantation. Additional analyses and comparisons of the secondary placement of the XEN stent to the Ahmed glaucoma valve or Baerveldt glaucoma implant are beyond the original scope of this study, but we do look forward to addressing this question in the future.

Most of our patients received XEN alone, but one received XEN combined with cataract surgery and achieved a comparable, significant reduction in IOP and number of medications. A meta-analysis study published recently has shown that there is no significant difference in IOP lowering between XEN alone or combined XEN and cataract surgery [25]. In contrast, another study concluded that combined cataract surgery had a higher rate of failure [19]. This discrepancy may be attributed to disparities in glaucoma subtypes, including a mixture of primary and refractory glaucoma in different studies. Our patients did not report any complications that posed a threat to their vision or a subsequent need for needling or additional glaucoma procedures. Needling revision has been recognized as one of the risk factors for long-term surgical failure [26]. It is commonly used to restore the functionality of failed filtering blebs due to bleb fibrosis, which can occur in up to 45% of cases [27]. It is possible that our sample size is small and follow-up period is not long enough to detect subconjunctival fibrosis around a XEN implant. However, we believe that that the tenonectomy procedure and the use of a large volume of MMC (0.3 mL of 0.4 mg/mL) to reduce subconjunctival fibrosis were associated with a high rate of successful treatment outcomes.

## Conclusion

This research reported a successful XEN stent implantation after a failed GDD. Implantation of an XEN Gel stent in conjunction with a large volume of mitomycin C and tenonectomy may be a viable surgical alternative for patients who have experienced a previous failure GDD and require additional IOP reduction. Given that GDD surgery is a common procedure, and that additional intervention may be required if the shunt provides insufficient IOP control, further long-term and large-population studies are warranted to investigate the impact of factors including gender, ethnicity, type of glaucoma, prior surgery, and combined or standalone procedures on the success rate of XEN implantation following a prior failed shunt tube.

## Data Availability

Data is provided within the manuscript. The datasets used and/or analysed during the current study are available from the corresponding author on reasonable request.

## Abbreviations

GDD: Glaucoma drainage device
IOP: Intraocular pressure
MIGS: Minimally invasive glaucoma surgeries
MMC: Mitomycin C
